# Gene editing isn’t just about food: comments from U.S. focus groups

**DOI:** 10.1080/21645698.2021.1919485

**Published:** 2021-05-20

**Authors:** Brandon R. McFadden, Joy N. Rumble, Kathryn A. Stofer, Kevin M. Folta, Savanna Turner, Adam Pollack

**Affiliations:** a Department of Applied Economics and Statistics, University of Delaware, Newark, DE, USA; b Department of Agricultural Communication, Education, and Leadership, The Ohio State University, Columbus, OH, USA; c Department of Agricultural Education and Communication, University of Florida, Gainesville, FL, USA; d Horticultural Sciences Department, University of Florida, Gainesville, FL, USA

**Keywords:** Genome editing, science communication, disease, designer babies, food

## Abstract

In the United States, adult public perception of genetic modification has been well documented in the domain of agriculture and food; however, recent international news on gene editing in medical applications may present new challenges for science communicators who seek to proactively share benefits of emerging gene editing technology. While research traditionally considers perceptions of agricultural and medical applications separately, gene editing may bridge the gap between the two domains. We find that when asked about thoughts regarding gene editing, adult focus groups discussed medical applications more frequently and extensively than agricultural applications. Although, when examining the length of discussion about specific topics, designer babies, cures for disease, and food were discussed at similar lengths. Understanding audiences’ current perceptions of the technology is the first step in shaping strategic communication efforts to inform public opinion. A proper understanding of the benefits and risks of new technology is central to its application.

## Introduction

Although human insulin was the first product from a genetically modified organism (GMO) approved by the U.S. Food and Drug Administration for human consumption in 1982,^1^ the vast majority of literature examining public perceptions about GMOs has focused on food and food ingredients.^2–5^ It is not clear why research has focused more on agricultural applications, but it may be because public attention and opposition to GMOs are more pronounced for food compared to medical applications. However, this focus may shift for gene editing applications.

A video uploaded to YouTube on November 25, 2018, announced the birth of twins whose genes were altered, using gene editing technology, to prevent HIV infection and viral reproduction (https://youtu.be/th0vnOmFltc). This represented a milestone in medicine, a new quandary for bioethicists, and a shocking realization that engineering DNA-level health outcomes in humans were now a reality. The story rapidly became international news^6^ and an analysis of Google metrics showed that public interest in the science behind the immune twins surged upon announcement (Google Trends), introducing a curious and potentially concerned public to the possibilities of gene editing.

Rapid awareness of a new and complex technology with serious ethical considerations presents new challenges for related industries, policymakers, and science communication specialists. The media announcement about human editing likely implied haphazard application to some, if not many, and may have fostered misunderstanding about gene-editing technology.^7^ Until the announcement, most public exposure to genetic-engineering concepts has centered around agricultural applications.^8^

The reality of gene-edited humans raised the question of how the public would respond to the technology and whether the focus of genetic-engineering techniques will shift from agricultural to medical applications with these latest developments. It is not currently clear whether individuals link gene editing with human health or food, or both, and whether support in one domain will assist or hinder support in the other. Connections between gene editing applications developed for agricultural and medical uses may shift public concerns and create a new challenge for science and health communicators.

Personal values and beliefs associated with food and human health shape perception of agricultural and medical technologies.^9^ If the applications of gene editing connect with values and beliefs, attitudinal conclusions about the science are likely to be favorable. If the characteristics of the gene editing application appear contrary to existing values and beliefs, conclusions about the science are likely to be unfavorable.^9^ However, individual attitudes about scientific issues are not static and can be influenced by media, politics, involvement, social contacts, and advocacy groups (NASEM, 2107). It is likely that a complex communication environment will exist around these topics and that a systems perspective, which considers transdisciplinary thinking across medicine and agriculture, and longitudinal research will be needed to fully understand how best to communicate about gene editing technologies in agriculture and medicine. Clear strategies will be needed to inform the public of emerging technologies, as well as to advocate for their implementation in response to a health crisis like the COVID19 pandemic.

Research examining public perception of genetically engineered humans is scant. Current research on the perceptions of genetically engineered humans that exists blurs the distinct difference between transgenesis – an addition or suppression of an entire gene or genes from heterologous species – and gene editing – a process that alters an extremely specific portion of the genetic code^10^ with limited, detectable off-target effects. Public support for gene-edited therapies in humans has been the focus of previous research; however, results about the level of public support are mixed.^11–13^ Evidence indicates that people are generally more supportive of medical treatments and risk reductions than enhancement or alteration of physical traits, and support for genetic intervention increases with the potential perceived severity of specific diseases.^14,15^ Trust in the enterprise of science as a whole,^16,17^ or experience in agriculture or medical contexts in particular can also affect public attitudes toward and support for the technology,^18,19^ and scientific issues that are contested and widely debated at the policy level draw more public opposition.^9^

This paper presents results from adult focus group participants from cities in four U.S. regions that query public interpretation of term “gene editing.” We analyzed conversations to identify the frequency and extent of comments around major themes: Human Health/Medical, Food and Agriculture, and Other, which were values-based concerns such as “Playing God” or “Not Natural.” We examined differences across the major themes along with other terms we classified as subthemes. Lastly, we then examined heterogeneity in the range of comments for the central themes across focus groups.

## Materials and methods

### Data collection

In Fall 2019, focus groups were conducted in a U.S. city for each of the four regions identified by the U.S. Census Bureau: Midwest, South, Northeast, and West. Data were collected from two focus groups each in Columbus, OH (Midwest), Dallas, TX (South), Philadelphia, PA (Northeast), and San Francisco, CA (West), for eight total focus groups. Information about participant characteristics for each location are presented in Appendix A1. In each location, the focus groups were held on a weeknight with the first group running from 5:30 to 7:30pm and the second focus group running from 8:00 to 10:00pm. All focus groups were held at an external research facility. The participants were provided light refreshments and a Visa gift card for their participation. The incentive for participants in Columbus, Dallas, and Philadelphia was 100. USD San Francisco participants received an incentive of 125 USD to account for a higher cost of living.

Ten to twelve participants were recruited for each focus group by an external marketing firm. The marketing firm used a recruitment script to screen and qualify participants. The script screened participants to ensure that they: 1) were a resident of the state; 2) had a smart phone; 3) had a neutral to positive trust in science; 4) had the ability to contribute thoughtful articulations; and 5) had not recently participated in other research. If the individuals did not meet all of these qualifications, they were not invited to participate in the study. Participants were not screened based on knowledge of or attitudes about gene editing. In addition to these qualifications, the marketing firms recruited participants for each group to include both males and females, a variety of ages (18 and older), income and education levels, and variety of races and ethnicities.

Some of the recruited participants were no-shows. A total of 64 participants participated in the study with group size ranging from 4 to 9, with an average of 8 participants in each group. The number of participants in each group was as follows: Philadelphia *n* = 18 (group 1: *n* = 9, group 2: *n* = 9); Dallas *n* = 16 (group 1: *n* = 8, group 2: *n* = 8); Columbus *n* = 17 (group 1: *n* = 9, group 2: *n* = 8); and San Francisco *n* = 13 (group 1: *n* = 4, group 2: *n* = 9).

**Figure 1.**
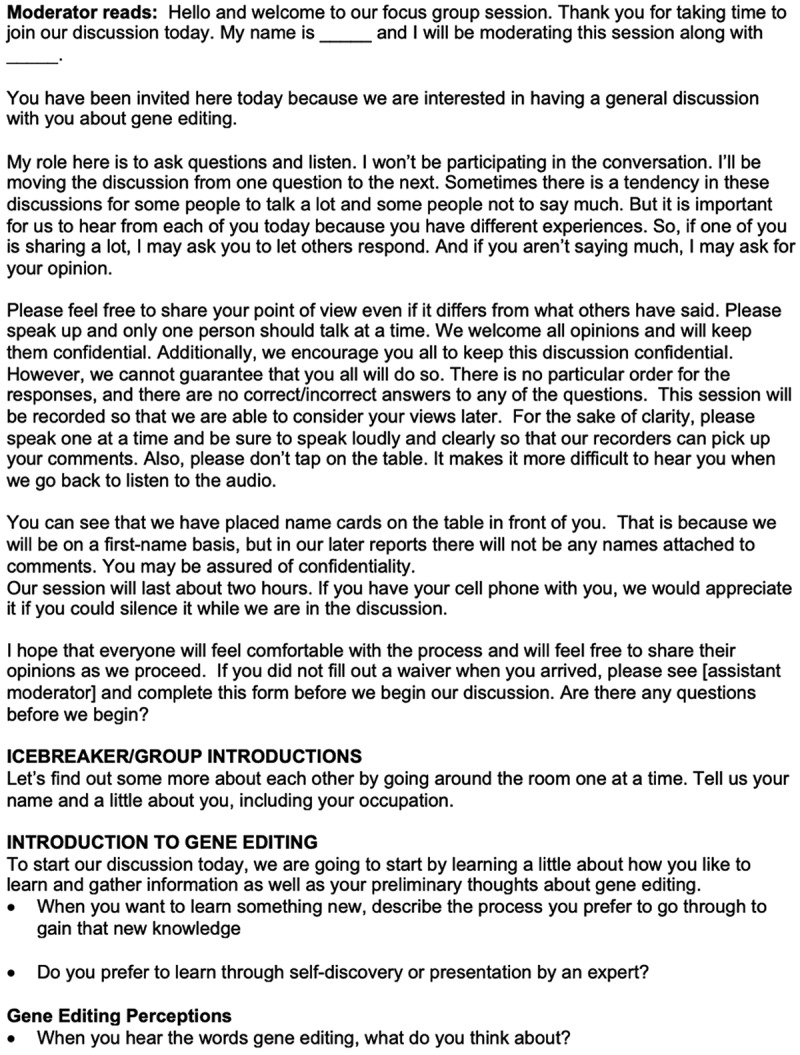
Moderator guide used to elicit perceptions about gene editing.

A semi-structured moderator’s guide was developed to guide each focus group discussion and ensure that data were gathered uniformly across focus groups. The moderator’s guide was developed primarily by two researchers with a focus on agricultural communication and education. The moderator’s guide was validated by a team of researchers with expertise in science communication, social implications of technology, and genetic alterations prior to use. The discussions were video recorded and transcribed for data analysis by the marketing firm. The portion of the moderator guide relevant to this study is shown in Figure 1. Focus groups began with an introduction to the moderators and instructions. An icebreaker activity was used to begin the conversation among participants and as an introduction of the participants. Prior to discussing gene editing, participants were asked about learning styles to spur engagement. Specifically, participants were asked: “When you want to learn something new, describe the process you prefer to go through to gain that new knowledge?” and “Do you prefer to learn through self-discovery or presentation by an expert?” Then, without prompting or priming about gene editing, participants were asked: “When you hear the words gene editing, what do you think about?” This is the question relevant to this study and used for data analysis. The portion of the discussions of interest in this study was led by a sole researcher specializing in agricultural communication in all eight focus group locations.

### Data analysis

When interpreting coded data from focus groups, an established framework is used to examine the frequency and extensiveness of comments.^20^ It is important to distinguish between frequency (i.e., number of comments made) and extensiveness of comments (i.e., word count) to better understand how often topics were discussed versus the length that topics were discussed.^21^ Therefore, we explored the transcribed data from the focus groups to determine frequency and word count. Further, to better understand the relationship between frequency and extensiveness of comments, a ratio was estimated (i.e., word count divided by frequency).

Based on initial review the data, comments were first coded and categorized into the *a priori* themes of Human Health/Medical, Food and Agriculture, and Other. After this initial separation the data within each theme were further analyzed for subthemes using open coding. Frequency of the themes and subthemes was determined through the coding process in MaxQDA. The data within each theme and subtheme were then exported to Microsoft Word to determine the word count of each data point. The unit of analysis was participant comments and observations made in the transcripts (e.g., laughter) and moderator quotes were not included in the word counts. An agricultural communicator led the coding process, and the themes and subthemes were confirmed by a larger multidisciplinary research team. Excerpts from the transcribed data are shown with associated subthemes in Appendix A2 to illustrate a comment (i.e., frequency), the extensiveness of a comment (i.e., word count), and how comments were categorized.

To determine differences in the frequency and extensiveness of comments across major themes, Chi-Square tests of independence for were estimated for frequency, word count, and the ratio of word count and frequency. If our null hypothesis of no difference across themes was rejected, pairwise comparisons of themes were estimated with Bonferroni corrected *P* values. Results from these tests provide some insight into what participants think about when hearing the term gene editing.

To explore the variation in extensiveness of comments across subthemes, Chi-Square tests of independence were estimated within a major theme and across major themes using the subthemes in the 50^th^ percentile of word count. These tests were conducted for word count only, and not for frequency, because of low expected cell counts for frequencies across subthemes. If a null hypothesis was rejected for either of the Chi-Square test, pairwise comparisons were estimated using Bonferroni corrected *P* values. Results from these tests provide some insight about what participants discussed the most in conversations about gene editing.

Comments within subthemes were also categorized as negative, neutral, or positive to provide an understanding about the sentiment of comments. Categorization occurred as follows: Negative – overall negative response or focused on identifying disadvantages or concerns, Neutral – overall indifferent response. Including providing a positive and negative statement in the same comment or when sentiment was unable to be identified, and Positive – overall positive comment or focused on identifying advantages or benefits.

Finally, we then examined heterogeneity in extensiveness of comments for major themes and top five subthemes across cities were focus groups were conducted. For each city, we estimated the proportion of word count for each major theme (e.g., word count for Human Health/Medical divided by the sum of word count for Human Health/Medical, Food and Agriculture, and Other). Using the proportion of word count allows us to normalize how much a theme was discussed for a given city (i.e., some cities had more participants and a higher word count in general and examining proportions accounts for those variations). This allowed us to estimate which cities discussed certain themes the most. We also did the same analysis for the five subthemes discussed the most. Results from these tests provide some insight about how conversations concerning gene editing vary by geographic region.

## Results

Three major themes were identified prior to data analysis: 1) Human Health and Medical, 2) Food and Agriculture, and 3) Other. Frequency, word count, and the ratio of word count and frequency for the major themes are shown in Table 1. There is a significant difference in frequency across themes (Chi-Square test statistic = 31.53, *P* value <.001). Pairwise comparisons indicate that the frequency for Human Health/Medical was greater than both Food and Agriculture (Bonferroni-Corrected *P* value <.001) and Other (Bonferroni-Corrected *P* value <.001) themes. There was no difference between the frequency for Food and Agriculture and Other (Bonferroni-Corrected *P* value <.880).

**Table 1.** Frequency and word count for major themes discussed by participants

Themes	Frequency		Frequency
Human Health/Medical	52.0^a1^	1,362.0^a2^	26.19^a3^
Agriculture/Food	24.0^b1^	686.0^b2^	28.58^a3^
Other	18.0^b1^	185.0 ^c3^	10.28^b3^
Total	94.0	2,184.0	

Letters next to word counts represent pairwise comparison grouping within a major theme. Pairwise comparisons were conducted using Bonferroni corrected *P* values with a threshold of 0.01.

There is also a significant difference in word count across themes (Chi-Square test statistic = 1406.16, *P* value <.001). Pairwise comparisons indicate that word counts were significantly different across all multiple comparisons: word count for Human Health/Medical was greater than the other two themes (both Bonferroni-Corrected *P* values <.001), and word count for Food and Agriculture was greater than Other (Bonferroni-Corrected *P* value <.001).

Furthermore, there is a significant difference in the ratio word count and frequency across themes (Chi-Square test statistic = 13.70, *P* value = .002). Pairwise comparisons with Bonferroni corrected *P* values indicate that both Human Health/Medical (Bonferroni-Corrected *P* value = .006) and Food and Agriculture (Bonferroni-Corrected *P* value = .001) were greater than Other. However, there was no difference between the ratio for Human Health/Medical and Food and Agriculture (Bonferroni-Corrected *P* value = 1). Thus, the extensiveness of comments was similar when participants expressed a view about Food and Agriculture or Human Health/Medical.

The subthemes identified under the three major themes were: 1) Human Health/Medical – Designer Babies, Cures for Disease, Long-Term Effects (or need for long-term research), Eugenics, Medical Research, Medical Improvements, Genetics/DNA, CRISPR in the Medical Field, Human Health, and Diseases; 2) Food and Agriculture – GMOs, Cloning, Food, Monsanto, Seeds, Farm Raised Fish, and Agriculture; and 3) Other – Science Fiction, Play God, Altering Nature, Modern Science, and Positive Attitude (without an attachment to any application).

Word counts and sentiment for the subthemes are shown in Tables 2, 3, and 4. There were variations in the extensiveness of comments across subthemes within all themes (Chi-Square test statistic = 1,595 (Human Health/Medical), 1,109 (Food and Agriculture), 66 (Other), all *P* values <.001). The subthemes Cures and Designer babies had the highest word count for Human Health/Medical; however, more than half of the comments about Cures were generally positive, while more than half of the comments about Designer Babies were negative. Participants were most positive when commenting on Medical research, in general. Food had the highest word count across subthemes within Food/Agriculture. None of the subthemes within Food/Agriculture had an overall positive sentiment; indeed, half of the comments were negative for Food and GMOs. The subthemes Playing God and Science Fiction had the highest word count for Other, and comments within this overall theme were generally negative (note that there is not a sentiment for Positive Attitude because it was implicitly positive).

**Table 2.** Subtheme word count and sentiment for human health/medical

Theme Subthemes		Sentiment
Human Health /Medical	1,362.0	30% Negative, 47% Neutral, 23% Positive
Cures for Disease	364.0^A1^	56% Positive, 44% Neutral
Designer Babies	383.0^A1^	67% Negative, 22% Neutral, 11% Positive
Long Term Effects	280.0^B1^	56% Positive, 33% Neutral, 11% Positive
Eugenics	99.0^C1^	57% Negative, 43% Neutral
Medical Research	86.0^C1^	100% Positive
Medical Improvements	47.0^D1^	33% Neutral, 67% Positive
Genetics/DNA	49.0^D1^	50% Neutral, 50% Positive
CRISPR in the Medical Field	38.0^D1^	100% Neutral
Human Health	13.0^E1^	50% Neutral, 50% Positive
Disease	3.0^E1^	100% Negative

Letters next to word counts represent pairwise comparison groupings of subthemes within a major theme. Pairwise comparisons were conducted using Bonferroni corrected *P* values with a threshold of 0.01.

**Table 3.** Subtheme word count and sentiment for agriculture/food

Theme Subthemes	Word Count	Sentiment
Agriculture/Food	686.0	33% Negative, 58% Neutral, 8% Positive
Food	352.0^A2^	50% Negative, 25% Neutral, 25% Positive
GMOs	161.0^B2^	50% Negative, 50% Neutral
Seeds	87.0^C2^	33% Negative, 33% Neutral, 33% Positive
Cloning	50.0^D2^	20% Negative, 80% Neutral
Monsanto	8.0^E2^	25% Negative, 75% Neutral
Farm Raised Fish	17.0^E2^	100% Neutral
Agriculture	11.0^E2^	100% Neutral

Letters next to word counts represent pairwise comparison groupings of subthemes within a major theme. Pairwise comparisons were conducted using Bonferroni corrected *P* values with a threshold of 0.01.

The grouping of subthemes occurring in the top 50^th^ percentile of word counts is shown in Table 5. While the theme Human Health/Medical dominates overall conversations in both frequency and word count (Table 1), some nuance is detected when examining variation across word count for the subthemes in the 50^th^ percentile (Chi-Square test statistic = 1129.4003, *P* value < .001). Such that, the length of conversations about the subtheme Food, which was only the third most frequently mentioned subtheme in Food and Agriculture – 4 mentions – had a word count of approximately 350, which was similar to the two subthemes for Human Health/Medical with the highest frequency (i.e., Designer Babies – 10 mentions – and Cures for Disease – 9 mentions). Other subthemes in the 50^th^ percentile were Long-Term Effects, GMOs, Eugenics, Seeds, Medical Research, Playing God, Cloning, and Genetics/DNA.

Heterogeneity in word count, as a proportion of the overall discussion, for major themes and the top five subthemes across cities were focus groups were conducted are shown in Tables 4 and 5.

**Table 4.** Subtheme word count and sentiment for other

Theme Subthemes		Sentiment
**Other**	**185.0**	**71% Negative, 18% Neutral, 12% Positive**
Play God	71.0^A3^	75% Negative, 25% Neutral
Science Fiction	46.0^AB3^	100% Negative
Altering Nature	30.0^BC3^	100% Negative
Modern Science	21.0^C3^	50% Neutral, 50% Positive
Positive Attitude	17.0^C3^	

Letters next to word counts represent pairwise comparison groupings of subthemes within a major theme. Pairwise comparisons were conducted using Bonferroni corrected *P* values with a threshold of 0.01.

**Table 5.** Word count groupings for subthemes in the 50^th^ percentile

Subthemes		Major Theme
Designer Babies	383.0^a^	Human Health/Medical
Cures for Disease	364.0^a^	Human Health/Medical
Food	352.0^a^	Agriculture/Food
Long Term Effects	280.0^b^	Human Health/Medical
GMOs	161.0^c^	Agriculture/Food
Eugenics	99.0^d^	Human Health/Medical
Seeds	87.0^de^	Agriculture/Food
Medical Research	86.0^de^	Human Health/Medical
Play God	71.0^de^	Other
Cloning	50.0^e^	Agriculture/Food
Genetics/DNA	49.0^e^	Human Health/Medical

Letters next to word counts represent pairwise comparison groupings of subthemes across major themes. Pairwise comparisons were conducted using Bonferroni corrected *P* values with a threshold of 0.01.

As shown in Table 6, Human Health/Medical was discussed most in Dallas and the least in Columbus; there was not a difference between Philadelphia and San Francisco (*P* value < .888). Food and Agriculture was discussed most in Columbus and least in Dallas; again, there was not a difference between Philadelphia and San Francisco (*P* value < .117). Other was discussed most by Dallas and San Francisco, no difference (*P* value < .568), and least by Columbus and Philadelphia, no difference (*P* value < .629). For sub-themes (Table 7), Designer Babies was discussed most by Dallas and least in San Francisco; there was not a difference between Columbus and Philadelphia (*P* value = .213). Dallas discussed Cures for Disease the most and San Francisco was not far behind, then Philadelphia, and then Columbus. Columbus, Philadelphia, and San Francisco all spoke about Food at similar rates (Columbus and Philadelphia, *P* value = .249; Columbus and San Francisco, *P* value = .679; Philadelphia and San Francisco, *P* value < .213), and Dallas did not talk about topics in the sub-theme Food at all. Columbus spoke the most about GMOs and Dallas and San Francisco discussed GMOs at similar rates (*P* value = .029).

**Table 6.** Proportions of word count for major themes by city

	Theme		
City	Human Health/Medical	Agriculture/Food	Other
Columbus	0.461^c1^	0.469^a2^	0.070^b3^
Dallas	0.830^a1^	0.043^c2^	0.127^a3^
Philadelphia	0.699^b1^	0.238^b2^	0.063^b3^
San Francisco	0.651^b1^	0.234^b2^	0.114^a3^

Letters next to proportions represent pairwise comparison groupings within a major theme across cities. Pairwise comparisons were conducted using Bonferroni corrected *P* values with a threshold of 0.01.

**Table 7.** Proportions of word count for the top five subthemes by city

	Theme				
City	Designer Babies	Cures for Disease	Food	Long Term Effects	GMOs
Columbus	0.220^c1^	0.114^d2^	0.254^a3^	0.193^b4^	0.220^a5^
Dallas	0.477^a1^	0.513^a2^	0^b3^	0^c4^	0.010^b5^
Philadelphia	0.306^c1^	0.205^b2^	0.285^a3^	0.205^b4^	0^c5^
San Francisco	0.009^d1^	0.409^b2^	0.240^a3^	0.262^a4^	0.080^b5^

Letters next to proportions represent pairwise comparison groupings of subthemes across cities. Pairwise comparisons were conducted using Bonferroni corrected *P* values with a threshold of 0.01.

## Discussion

Focus group conversations about gene editing were mostly focused on medical applications, yet agricultural applications were also discussed. The focus on medical applications may be reflective of the participants’ awareness, spurred by media coverage like the gene edited, HIV-resistant babies ^[6,22,23]^ apparent cures for sickle cell disease,^24^ and impact on genetic diseases such as cystic fibrosis.^25^ Topics discussed outside of direct agricultural and medical applications could be described as ethical and moral considerations connected to gene editing, supporting participants’ use of values and beliefs to make sense of and interpret an abstract scientific topic. Such use of values is consistent with prior research.^9^ Our results also confirm the relevance of previous gene-therapy research that focused on public perceptions of altering physical traits versus medical treatments,^14,15^ as the most discussed topics were designer babies and cures for disease.

Designer babies, cures for disease, and food were the topics most extensively discussed topics by participants. While most discussion focused on medical applications, gene editing is also closely related to food in the minds of the American adult population with general trust in science. Still, participants in this study primarily associated gene editing with its medical applications. It is not yet clear how, or if, the association between gene editing and human health will affect perceptions of gene editing and agriculture. New breakthroughs that straddle the disciplines of medicine and agriculture, such as the GalSafe pig, edited to reduce sensitivity to a rare reaction to specific cell-surface sugars,^26^ may shift consumer sentiment by uniting medical and agricultural applications. Additionally, personal experience with agriculture or medicine could influence attitudes toward the technology in each domain, as previous research suggests may be the case with genetic engineering.^19^

There is an opportunity to examine the complex communication ecosystem around gene editing from a transdisciplinary systems perspective, bridging research and application in health and agriculture, as perceptions of gene editing are likely to change as the applications and issues evolve.^27^ Concurrent applications of gene editing in human health and agriculture further complicate this communication environment for genetic-engineering techniques. A collaborative transdisciplinary systems approach could more directly assess individual opinions of gene editing in medical vs agricultural contexts, compare opinions to expertise in each domain, probe trust in media and government agencies, and compare various terminology in common and technical use. Our study contributes to the literature by aiding researchers in understanding what application individuals generally trusting of science default to when they hear the term ‘gene editing’ and their sentiment toward those applications. Further research, including experimental communication studies, longitudinal assessments that track traditional and social media, and audience analyses, could provide a more robust understanding about the relationship between attitudes toward gene-edited medical and agricultural applications.

## Appendix A1. Characteristics of Focus Group Participants

**Table:**

**Characteristic**	Northeast	South	Midwest	West
Age	48.22 (40.67, 55.78)	53.88 (44.49, 62.26)	57.24 (49.27, 65.20)	52.00 (41.09, 62.91)
Education				
Less than high school	-	-	-	-
High school/GED	0.278 (0.067, 0.489)	-	-	0.154 (0.000, 0.354)
Some college/ trade school	0.278 (0.067, 0.489)	0.500 (0.250, 0.750)	0.471 (0.229, 0.713)	0.385 (0.115, 0.654)
Bachelor’s degree	0.278 (0.067, 0.489)	0.438 (0.190, 0.685)	0.353 (0.121, 0.585)	0.385 (0.115, 0.654)
Post-grad or higher	0.167 (0.000, 0.342)	0.063 (0.000, 0.183)	0.176 (0.000, 0.361)	0.077 (0.000, 0.225)
Ethnicity/Race				
White/ Caucasian	0.611 (0.381, 0.841)	0.563 (0.315, 0.810)	0.588 (0.350, 0.827)	0.538 (0.262, 0.815)
Black/African American	0.389 (0.159, 0.619)	0.375 (0.133, 0.617)	0.294 (0.073, 0.515)	0.077 (0.000, 0.225)
Hispanic/ Latino	-	-	-	0.154 (0.000, 0.354)
Asian	-	0.063 (0.000, 0.183)	-	-
Other	-	-	0.118 (0.000, 0.274)	0.231 (0.000, 0.464)
Female	0.556 (0.322, 0.790)	0.375 (0.133, 0.617)	0.529 (0.287, 0.771)	0.385 (0.115, 0.654)
Income				
Under $30,000	0.167 (0.000, 0.342)	0.125 (0.000, 0.290)	0.059 (0.000, 0.173)	0.231 (0.000, 0.464)
$30,000 – $69,999	0.500 (0.264, 0.736)	0.313 (0.081, 0.544)	0.412 (0.173, 0.650)	0.385 (0.115, 0.654)
$75,000 or more	0.333 (0.141, 0.359)	0.563 (0.315, 0.810)	0.529 (0.287, 0.771)	0.385 (0.115, 0.654)
N	18	16	17	13

Means are reported for Age, and proportions are reported for the other variables. Number in parentheses are the upper and lower confidence limits for the 95% confidence interval (lower confidence limits with negative values were censored at zero).

## Appendix A2. Excerpts from Transcribed Data and Associated Subthemes

**Table:**

Human Health/Medical Subthemes	Excerpts
Cures for Disease	I think about diseases and problems that people can have down the line. They can combat them early. That’s a benefit, I think.
Designer Babies	But I don’t want, you know, if you decide you want your baby to have blue eyes, or whatever, I don’t think you should go in and try, and edit the genes.
Long Term Effects	I was agreeing with definitely want it to be tested long-term, just for the side effects and so that no Frankenstein outcome comes of it.
Eugenics	No, it’s like what Hitler was trying to.
Medical Research	I think cancer research.
Medical Improvements	Just medical improvements in general.
Genetics/DNA	I guess, you can say genetics, I guess.
CRISPR in the Medical Field	Lately, I also think of the CRISPR thing, because it’s been in the news a lot, and technology being used for things that weren’t previously used for or things that are debated in the medical field ethics-wise.
Human Health	I jump between do I like it in humans or agriculture.
Disease	I think cancer.
**Agriculture/Food Subthemes**	**Excerpts**
GMOs	I even avoid GMO foods. I won’t buy foods that are genetically modified, and I stay away from corn and so forth. I will not consume those. It concerns me.
Cloning	Or like cow cloning. Yeah, they do that, too. Sheep.
Food	I hope I’m not being awkward, but as far as gene editing, I was at a barbecue and somebody had a chicken wing. This was the chicken leg. Somebody else had another chicken leg this – I’m like, “What the heck’s going on here? How big’s your chicken? Where is a chicken that was that big?” It’s the editing of the genes or whatever they’re putting in the genes and I contribute that to what’s going on now.
Monsanto	Evil Monsanto.
Seeds	I was talking about because I watched a documentary in my environmental science class last fall, and basically, it was about seeds – I forget what kind of seeds – but they would genetically modify the seeds and they would patent the genetically modified seeds, and then they would require companies and farmers and whoever else to buy new seeds every year for re-implantation, and they just took over the whole industry within a decade or so.
Farm Raised Fish	Yeah. Like farm – like the salmon or tilapia, all that farmed – Farm-raised.
Agriculture	I jump between do I like it in humans or agriculture.
**Other** **Subthemes**	**Excerpts**
Science Fiction	The whole Jurassic Park.
Play God	No, I’m just wondering how much we play God.
Altering Nature	Modifying or altering nature.
Modern Science	I just think modern science when I hear that term.
Positive Attitude	I like to think of it as a positive, too, instead of a – what was that? Dolly?
